# Is sexual autonomy a protective factor against intimate partner violence? Evidence from 27 sub-Saharan African countries

**DOI:** 10.1371/journal.pone.0308108

**Published:** 2024-07-29

**Authors:** Getayeneh Antehunegn Tesema, Fred Yao Gbagbo, Sylvester R. Okeke, Edward Kwabena Ameyaw, Sanni Yaya

**Affiliations:** 1 Department of Epidemiology and Biostatistics, College of Medicine and Health Sciences, University of Gondar, Gondar, Ethiopia; 2 Department of Health Administration and Education University of Education Winneba, Kumasi, Ghana; 3 Centre for Social Research in Health, UNSW Sydney, Kensington, Australia; 4 Institute of Policy Studies and School of Graduate Studies, Lingnan University, Tuen Mun, Hong Kong; 5 L & E Research Consult Ltd, Wa, Upper West Region, Ghana; 6 The George Institute for Global Health, Imperial College London, London, United Kingdom; NUST: National University of Sciences and Technology, PAKISTAN

## Abstract

**Background:**

Though women in sub-Saharan Africa have increased risk of intimate sexual violence, research on the association between sexual autonomy and intimate partner violence among this population has not received the requisite attention. Consequently, we investigated if sexual autonomy is a protective factor against intimate partner violence among women in sub-Saharan Africa.

**Methods:**

Secondary data analysis was conducted based on the Demographic and Health Surveys (DHSs) of 27 sub-Saharan African countries from 2008 to 2021. A total of 104,523 married or cohabitating women were included in the study. We applied a multilevel Poisson regression model with robust variance to identify associated factors. Variables with a p-value<0.2 in the bi-variable multilevel Poisson regression analysis were considered for the multivariable analysis. The Adjusted Prevalence Ratio (APR) with its 95% confidence interval (CI) was reported, and variables with a p-value <0.05 were included in the multivariable analysis.

**Results:**

The prevalence of intimate partner violence and sexual autonomy among women in SSA were 32.96% [95% CI: 32.68%, 33.25%] and 88.79% [95% CI: 88.59%, 88.97%], respectively. Women in Sierra Leone had the highest prevalence of IPV (52.71%) while Comoros had the lowest prevalence of IPV (8.09%). The prevalence of sexual autonomy was highest in Namibia (99.22%) and lowest in Mali (61.83%). The MOR value in the null model was 1.26. We found that women who had sexual autonomy are 1.28 times [APR = 1.28, 95% CI: 1.17, 1.40] more likely to experience IPV than women who had no sexual autonomy.

**Conclusion:**

This study has demonstrated that sexual autonomy is significantly associated with intimate partner violence, however, it does not necessarily act as a protective factor. The study suggests the need for more education on intimate partner violence targeting women’s partners. This can help secure the commitment of the perpetrators to rather become proponents of anti-intimate partner violence and further offer women the necessary support for them to attain their full fundamental rights in all spheres of life.

## Background

Intimacy is conceived as a unitary construct of only one component [1]. Over the years, this perception has evolved due to variations in social exchange between intimate and other relationships [2], as sexual autonomy among women has gained a prominent stance in various conventions to protect the sexual and reproductive rights of women [3]. This evolution has demonstrated how intimacy could be multi-faceted covering about nine separate subcomponents thereby making it imperative for partners to meet the intimacy needs of their mates in all the nine areas to ensure optimal sexual satisfaction in intimate relationships [1].

Sexual autonomy may be explained as the ability to decline risky and non-risky sexual relations, as well as one’s ability to demand his/her partner to use a condom before intercourse whether in marital or non-marital relationships [4]. Sexually autonomous women are able to take control of their sexual life, including setting boundaries about who they should be intimate with, and are able to take initiatives to prevent sexually transmissible infections as well as unplanned pregnancies [5]. In the absence of women’s sexual autonomy, adverse sexual and reproductive health outcomes like unsafe abortions [6], unintended pregnancies [7, 8] sexually transmissible infections [9], and compromised access to effective modern contraception [10, 11] become prevalent.

Sexual autonomy is linked with women’s empowerment and has been conceptualized as a human rights issue. This is because sexual autonomy helps protect and maintain an informed decision over one’s body, one’s sexuality, and one’s sexual experience [12–14]. Evidence abounds that for a society to provide and promote women’s sexual autonomy, partner involvement cannot be overlooked as partner involvement increases awareness and recognition of women’s sexual autonomy [15, 16].

Intimate partner violence (IPV) permeates sub-Saharan Africa, with an average prevalence (36%) surpassing the global average of 30% [17]. IPV refers to the “physically, sexually, and psychologically harmful behaviours in the context of marriage, cohabitation, or any other form of union, as well as emotional and economic abuse and controlling behaviours” [18]. Globally, about 38%-50% of women’s murderers are their intimate partners [19]. The United Nations through its Sustainable Development Goals has identified IPV as a major threat to women’s wellness. Hence, the United Nations seeks to eliminate violence against females as enshrined in target 5.2 of the Sustainable Development Goals [20]. Besides, several international organisations have responded to IPV in diverse ways. For instance, in 1992, the Committee on the Elimination of Discrimination Against Women (CEDAW) indicated that violence against women is discriminatory and must be averted [21]. Beside the adverse physical and mental health consequences of IPV, it poses threat to women’s sexual and reproductive health [22–24].

Women in Africa are liable to lifetime partner violence (45.6%) and sexual assault (11.9%) than women in other parts of the world [17]. Research knowledge on the association between sexual autonomy and intimate partner violence among women in SSA is still emerging. Scholars have contributed to different aspects of this subject. For instance, in East Africa, Tessema et al. [25] explored the prevalence and factors associated with IPV. Their study revealed that at least 4 out of 10 of their sample had experienced IPV. Another study from East Africa also focused on the determinants of IPV and reported a prevalence of 32.6% [26]. The available studies on SSA tend to have focused on only the urban women [27], the association between access to water sources and IPV [28] and determinants of IPV [29, 30], among other issues. As to whether sexual autonomy is protective against IPV among SSA women in the reproductive age remains unexplored in the literature. Hence, there is a need to investigate this relationship among reproductive-aged women in the sub-region using the most current and representative datasets to ascertain how this nuanced perspective would expand the frontiers of knowledge on IPV.

Besides, considering the global agenda of achieving gender equality and empowering women and girls by 2030 [20], it is expedient to explore how a critical factor like sexual autonomy relates to IPV, in the context of SSA. Thus, the present study aims to extend this knowledge by investigating if sexual autonomy is a protective factor against intimate partner violence among women in SSA. Policy-wise, this study will point to areas that warrant policy interest by shedding light on how these two factors interrelate as well as how their relationship is shaped by women’s socio-demographic characteristics. Thus, we anticipate that this study will foreground impactful and well-tailored policy interventions needed to mitigate intimate sexual violence in SSA.

## Methods

### Data source and sampling procedure

Secondary data analysis was conducted based on the Demographic and Health Surveys (DHSs) of 27 sub-Saharan African countries from 2008 to 2021. DHS is a cross-sectional study that collects data to generate updated health and health-related indicators. A multistage stratified cluster sampling technique was employed to recruit the samples using Enumeration Areas (EAs) as primary sampling units and households as the secondary sampling units. Each country’s survey consists of men, women, children, birth, couple, and household datasets. The Individual Record (IR) dataset was used for this study after we obtained an authorization letter from the measure DHS program for data access. We extracted the data from the IR dataset based on literature and then appended using the STATA command "append using". Only women who were married or cohabitating were the denominators. The final sample size for this study was 104,523 women [Table 1].

**Table 1:** Description of the study sample.

Country	Year of survey	Weighted sample	Percentage (%)
Angola	2015–16	5,561	5.32
Burkina Faso	2010	9,431	9.02
Burundi	2016–17	6,445	6.17
DR Congo	2013–14	4,358	4.17
Cote d’Ivoire	2011–12	4,100	3.92
Cameroon	2018	3,917	3.75
Ethiopia	2016	3,905	3.74
Gabon	2012	3,113	2.98
Gambia	2019–20	1,586	1.52
Kenya	2014	3,512	3.36
Comoros	2012	1,797	1.72
Liberia	2019–20	1,629	1.56
Madagascar	2021	4,389	4.20
Mali	2018	3,043	2.91
Mauritania	2019–21	2,195	2.10
Malawi	2015–16	4,516	4.32
Mozambique	2015	1,884	1.80
Nigeria	2018	8,041	7.69
Namibia	2013	1,106	1.06
Rwanda	2019–20	1,693	1.62
Sierra Leone	2019	3,522	3.37
Sao Tome	2008–09	1,320	1.26
Chad	2014–15	1,886	1.80
Togo	2013–14	4,504	4.31
Uganda	2016	6,109	5.84
Zambia	2018	6,052	5.79
Zimbabwe	2015	4,907	4.69
**Total**	**-**	**104,523**	**100**

### Measurement of variables

#### Dependent variable

Having experienced IPV was the outcome variable for this study. Women were asked if they experienced any of the specified acts of physical, sexual, or emotional violence committed by their current husband/partner or most recent husband/partner in the past 12 months preceding the survey. Women who experienced any of the specified acts of physical, sexual or emotional violence were considered as experienced IPV, and if not were considered as never experienced IPV. In DHS, information was obtained from women who were married or cohabitating on their experience of violence committed by their current or former husbands/partners. Women were asked a series of questions that had four responses: never, often, sometimes, and yes but not the last 12 months, that is emotional spousal violence (say or do something to humiliate you in front of others; threaten to hurt or harm you or someone close to you; insult you or make you feel bad about yourself); physical spousal violence (push you, shake you, or throw something at you; slap you; twist your arm or pull your hair; punch you with his/her fist or with something that could hurt you; kick you, drag you, or beat you up; try to choke you or burn you on purpose; or threaten or attack you with a knife, gun, or any other weapon) and sexual spousal violence (physically force you to have sexual intercourse with him even when you did not want to; physically force you to perform any other sexual acts you did not want to; force you with threats or in any other way to perform sexual acts you did not want to) [31].

#### Independent variables

Informed by existing literature [29, 32–34], Socio-demographic variables, maternal characteristics, husband characteristics, and household and contextual-related variables were considered independent variables. However, the most important independent variable in this study was sexual autonomy. It was a composite variable created from three variables found in DHS; 1) “respondent can refuse sex?”, 2) “respondent can ask partner to use condom?” and 3) “wife is justified in asking the husband to use condom?”. Each variable has two responses “Yes" and "No" labelled as "1" and "0", respectively. Then, the three variables were added up together to create the variable sexual autonomy resulting in a score ranging from 0 to 3. To create the variable "sexual autonomy" respondents who answered "Yes" to at least one of the questions were considered as having sexual autonomy while those who answered "No" to the two questions were considered as not having sexual autonomy. Then a woman had a score of 0 labelled as "No" and those scored 1–3 were labelled as "Yes" for sexual autonomy.

Other independent variables included in the study were maternal age (< 20 years, 20–29 years, and ≥ 30 years), maternal educational status (No, Primary, secondary, and higher), husband age (<30 years, 30–39 years, 40–49 years and ≥ 50 years), media exposure (No and Yes), wealth status (poorest, poorer, middle, richer and richest), husband education (No, Primary, Secondary and Higher), sex of household head (Male and Female), marital status (Married and Cohabitating), maternal employment status (Working and Not working), residence (Urban and Rural), distance to health facility (Big problem and Not a big problem), and parity (nulliparous, 1–4 and ≥5). Media exposure was calculated by aggregating three variables such as watching television, listening to the radio, and reading newspapers. Then categorized as having media exposure if a mother has been exposed to at least one of the three and not if she had no exposure to any of the media sources.

#### Data analysis

The statistical analysis was a two-step procedure. First, we have fitted a multilevel modified Poisson regression to identify factors significantly associated with IPV including sexual autonomy. Secondly, to assess the role of sexual autonomy and other modifiable significant factors in determining IPV, we estimated the Population Attributable Fractions (APF). APF was estimated after fitting the multilevel modified Poisson regression model using post estimation command “punaf”.

To adjust for the non-response and sampling design, the data were weighted using the weighting variable. Stata version 17 statistical software was used for data management and analysis. Since the DHS data has a hierarchical nature, women within the same cluster/country might share similar characteristics to women from different clusters. This could violate the assumptions of the traditional regression model; these are the independence of observations and equal variance assumptions. Therefore, a multilevel model was fitted to identify factors associated with IPV using the country as a random variable (since there was no significant clustering effect when we used EAs as a random variable). Intra-class Correlation Coefficient (ICC), and Median Odds Ratio (MOR) were computed to measure the variation between clusters. The ICC quantifies the degree of heterogeneity between clusters (the proportion of the total observed individual variation in IPV among women is attributable to between cluster variations).

ICC = ϭ^2^/ (ϭ^2^+π^2^/3) [35], but MOR quantifies the variation or heterogeneity in outcomes between clusters and is defined as the median value of the odds ratio between the cluster at more likely to experience IPV and cluster at lower risk when randomly picking out two clusters (country).

$$\text{MOR}=\text{exp}(\;\sqrt{2*\partial 2*0.6745})∼\text{MOR}=\text{exp}(0.95*\partial )$$

(Merlo et al [36]). *∂*^2^ indicates that cluster variance.

Besides, DHS was a cross-sectional study, and the prevalence of IPV among women in SSA was 33%, which was greater than 10%. In this scenario, reporting the odds ratio could exaggerate the relationship between the independent variables and IPV [37, 38]. Therefore, the prevalence ratio is the best measure of association for the current study, and we applied a multilevel Poisson regression model with robust variance to identify associated factors. We preferred this model for three reasons. For start, when the magnitude of the outcome variable is common, the odds ratio obtained using the binary logistic regression approach overestimates the strength of the relationship. Second, because the DHS data is hierarchical, mothers were nested within cluster/EA/country. The third reason was that the multilevel robust Poisson regression model outperformed the multilevel log-binomial regression model in terms of convergence. As a result, our model considers data dependencies as well as the problem of overestimation.

Variables with a p-value<0.2 in the bi-variable multilevel Poisson regression analysis were considered for the multivariable analysis. Deviance was used to verify model fitness, and a model with the lowest deviance (-2LLR [Log-likelihood Ratio]) was considered the best-fit model. Finally, the Adjusted Prevalence Ratio (APR) with its 95% confidence interval (CI) was reported, and variables with a p-value <0.05 in the multivariable analysis.

Attributable Fraction (PAF): The adjusted PR for sexual autonomy obtained from the final best-fitted model was used to estimate PAF. PAF is interpreted as the proportion of IPV that could be eliminated by removing or changing the distribution of the risk factor. They are mainly used to assess the relative importance of the risk factor and assist for prioritizing interventions. It was estimated as [1];

$$PAF=P_{r}(\frac{{PR}_{adj}-1}{{PR}_{adj}})$$

Where *PR*_*adj*_ is the adjusted prevalence ratio and *P*_*r*_ is the prevalence of IPV, *P*_*e*_ is the proportion of IPV cases exposed to the risk factor (sexual autonomy). These formulas provide unbiased estimations of the population-attributable risk in the presence of confounders.

#### Ethical consideration

There was no need for ethical clearance as the researcher did not interact with respondents. The data used was obtained from the MEASURE DHS Program, and permission for data access was obtained from the Measure DHS program through an online request from http://www.dhsprogram.com.

## Results

### Background characteristics of women in Sub-Sahara Africa

A total of 104,523 married and cohabitating women were included in the study. Of them, 67,922 (64.98%) were rural residents and 20,426 (19.54%) belonged to the poorest household. More than half (52.44%) of women aged 30 years and above, and about 38,241 (37.35) of their husbands aged 30–39. More than three-fourths (77.77%) of women were married and more than one-third (34.91%) attained primary education. About 66.58% and 66.80% had media exposure and were working, respectively (Table 2).

**Table 2:** Background characteristics of women in sub-Sahara Africa.

Variable	Weighted frequency	Percentage (%)
**Residence**		
Urban	36,601	35.02
Rural	67,922	64.98
**Household wealth status**		
Poorest	20,426	19.54
Poorer	21,206	20.29
Middle	21,075	20.16
Richer	21,500	20.57
Richest	20,316	19.44
**Maternal age (in years)**		
<20	6,547	6.28
20–29	43,076	41.29
≥30	54,709	52.44
**Partner’s age (in years)**		
<30	21,326	20.83
30–39	38,241	37.35
40–49	26,816	26.19
≥50	16,004	15.63
**Maternal educational status**		
No	36,252	34.68
Primary	36,491	34.91
Secondary	27,259	26.58
Higher	4,521	4.33
**Partner’s educational status (n = 99,403)**		
No	28,937	29.11
Primary	29,731	29.91
Secondary	32,454	32.65
Higher	8,281	8.33
**Maternal employment status**		
Working	69,778	66.80
Not working	34,686	33.20
**Media exposure**		
No	34,862	33.42
Yes	69,460	66.58
**Sex of household head**		
Male	87,557	83.77
Female	16,966	16.23
**Marital status**		
Married	81,283	77.77
Cohabiting	23,240	22.23
**Perceived distance to a health facility**		
A big problem	41,308	40.26
Not a big problem	61,283	59.74
**Parity**		
Nulliparous	6,505	6.24
1–4	65,208	62.50
≥5	32,619	31.26
**Smoking cigarette**		
No	99,828	99.09
Yes	915	0.91
**Intimate partner violence**		
No	70,071	67.04
Yes	34,452	32.96
**Sexual autonomy**		
No	11,720	11.21
Yes	92,803	88.79

### The prevalence of sexual autonomy and intimate partner violence in Sub-Sahara Africa

**Fig 1:**
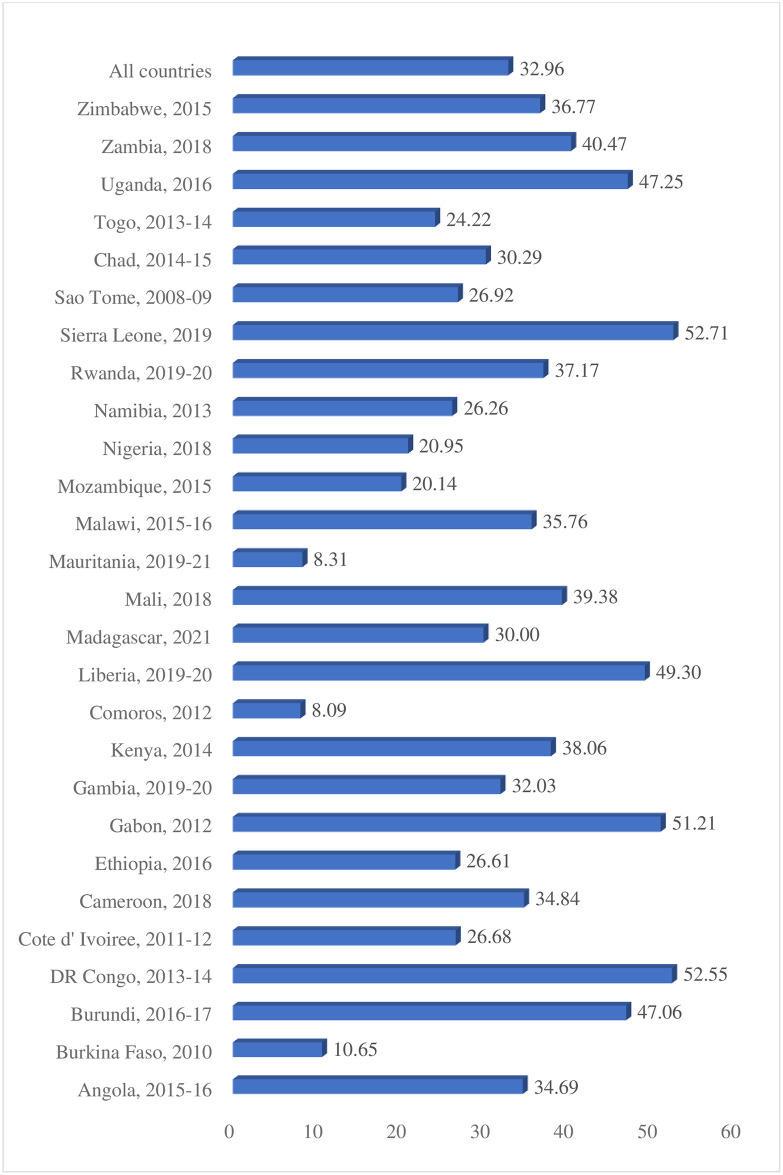
Prevalence of IPV among married and cohabitating women in Sub-Sahara Africa.

**Fig 2:**
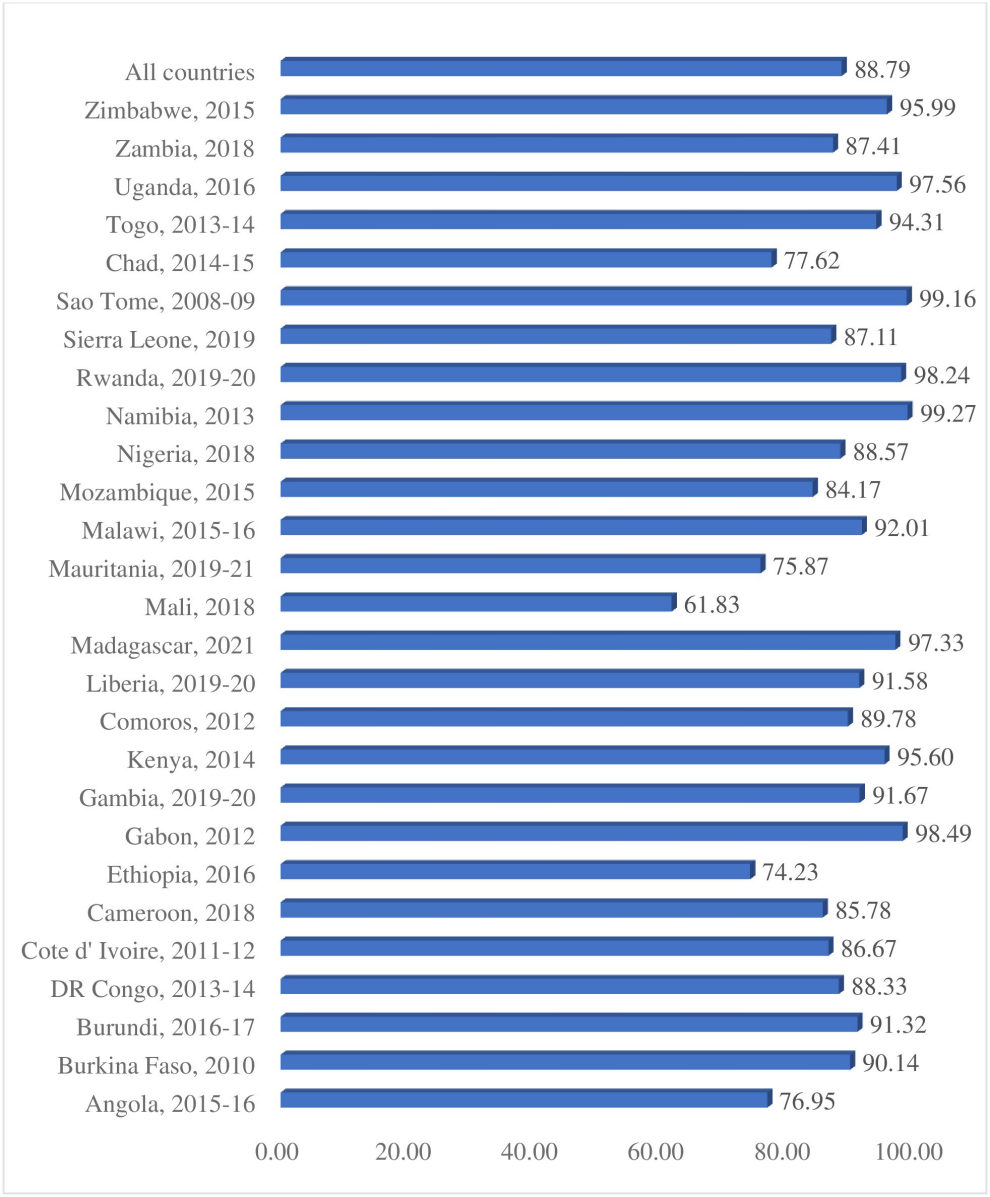
Proportion of married and cohabitating women who have sexual autonomy in Sub-Sahara Africa.

The prevalence of intimate partner violence and sexual autonomy among women in SSA were 32.96% [95% CI: 32.68%, 33.25%] and 88.79% [95% CI: 88.59%, 88.97%], respectively. Women in Sierra Leone had the highest prevalence of IPV (52.71%) while Comoros had the lowest prevalence of IPV (8.09%) [Fig 1]. The prevalence of sexual autonomy was highest in Namibia (99.22%) and the lowest was observed in Mali (61.83%) (Fig 2).

### Association between IPV among women and sexual autonomy and other related characteristics

We assessed the presence of a statistically significant association between IPV and independent variables. IPV had a statistically significant association with sexual autonomy, residence, wealth status, maternal education, husband education, maternal age, maternal education, perceived distance to health facility, maternal employment status, marital status, media exposure, and parity. The prevalence of IPV among women belonging to the richest and poorest households was 27.26% and 45.38%, respectively. Women aged 30 years and above (33.90%) had a higher prevalence of experiencing IPV than those aged under 20 years (24.79%) [Table 3].

**Table 3:** The distribution of intimate partner violence across sexual autonomy and other related characteristics of women.

Variable	Weighted frequency	IPV (%)	p-value
**Residence**			
Urban	36,601	31.99	<0.001
Rural	67,922	33.48	
**Household wealth status**			
Poorest	20,426	35.38	<0.001
Poorer	21,206	35.51	
Middle	21,075	33.96	
Richer	21,500	32.56	
Richest	20,316	27.26	
**Maternal age (in years)**			
<20	6,547	24.79	<0.001
20–29	43,076	33.08	
≥30	54,709	33.90	
**Partner’s age (in years) (n = 102,387)**			
<30	21,326	33.89	<0.001
30–39	38,241	34.22	
40–49	26,816	33.06	
≥50	16,004	30.09	
**Maternal educational status**			
No	36,252	28.53	<0.001
Primary	36,491	38.48	
Secondary	27,259	33.66	
Higher	4,521	19.74	
**Partner’s educational status (n = 99,403)**			
No	28,937	26.74	<0.001
Primary	29,731	38.82	
Secondary	32,454	36.02	
Higher	8,281	24.36	
**Maternal employment status**			
Working	69,778	34.91	<0.001
Not working	34,686	29.04	
**Media exposure**			
No	34,862	33.49	0.01
Yes	69,460	32.74	
**Sex of household head**			
Male	87,557	32.94	0.74
Female	16,966	33.86	
**Marital status**			
Married	81,283	30.76	<0.01
Cohabiting	23,240	40.66	
**Perceived distance to health facility (n = 102,591)**			
A big problem	41,308	34.77	<0.001
Not a big problem	61,283	32.14	
**Parity**			
Nulliparous	6,505	21.81	<0.001
1–4	65,208	32.53	
≥5	32,619	36.16	
**Smoking cigarette (n = 100,743)**			
No	99,828	33.12	<0.01
Yes	915	48.01	
**Sexual autonomy**			
No	11,720	26.10	<0.01
Yes	92,803	33.83	

### Multilevel robust Poisson regression analysis of sexual autonomy and IPV among women in Sub-Sahara Africa

The ICC value in the null model was 6.74%, indicating that about 6.74% of the overall variability of IPV was attributable to the between-cluster variability while the remaining 93.26% of the overall variability was due to the individual variation. In addition, the MOR value in the null model was 1.26, revealing that if we randomly transfer married or cohabitating women from the cluster with a lower likelihood of IPV to a cluster with a higher likelihood of IPV, would have a 1.26 times higher risk of experiencing IPV. As a rule of thumb, ICC>5% and MOR>1 are suggestive of the presence of a clustering effect in the data specifically when the outcome variable is not continuous and therefore, the multilevel models are appropriate.

In the multivariable multilevel robust Poisson regression analysis; sexual autonomy, parity, marital status, media exposure, maternal employment status, maternal educational status, maternal age, household wealth status, and residence were significantly associated with IPV. We found that women who had sexual autonomy had 1.28 times [APR = 1.28, 95% CI: 1.17, 1.40] the higher prevalence of experiencing IPV than women who had no sexual autonomy. The prevalence of IPV among rural resident women decreased by 7% [APR = 0.93, 95% CI: 0.89, 0.96] compared to urban women. The prevalence of experiencing IPV among women belonging to the middle, richer and richest households decreased by 8% [APR = 0.92, 95% CI: 0.88, 0.95], 12% [APR = 0.88, 95% CI: 0.83, 0.93], and 25% [APR = 0.75, 95% CI: 0.68, 0.85] compared to those belonged to poorest household, respectively.

Women aged 20–29 years and 30 years and above had 1.19 times [APR = 1.19, 95% CI: 1.12, 1.26] and 1.18 times [APR = 1.18, 95% CI: 1.09, 1.28] the higher prevalence of IPV compared to women aged under 20 years, respectively. Women who attained primary education were 1.09 times [APR = 1.09, 95% CI: 1.02, 1.18] the higher prevalence of IPV compared to women who didn’t have formal education while the prevalence of IPV among women who had higher education decreased by 34% [APR = 0.66, 95% CI: 0.60, 0.77] than women who didn’t have formal education. Women who were working had 1.14 times [APR = 1.14, 95% CI: 1.08, 1.20] the higher prevalence of experiencing IPV compared to women who were not working. Having media exposure increased the prevalence of experiencing IPV by 1.09 [APR = 1.09, 95% CI: 1.05, 1.14], and cohabitating women had 1.17 times [APR = 1.17, 95% CI: 1.07, 1.28] the higher prevalence of experiencing IPV compared to married women. Multiparous and grand multiparous women had 1.32 times [APR = 1.32, 95% CI: 1.22, 1.43] and 1.45 times [APR = 1.45, 95% CI: 1.33, 1.18] the higher prevalence of IPV compared to nulliparous women, respectively (Table 4).

**Table 4:** Multilevel robust Poisson regression analysis of factors associated with intimate partner violence among women in sub-Sahara Africa.

Variable	Crude Prevalence Ratio (CPR) with 95% CI	Adjusted Prevalence Ratio (APR) with 95% CI
**Residence**		
Urban	1	1
Rural	1.06 [0.89, 0.96]**	0.93 [0.89, 0.96]*
**Household wealth status**		
Poorest	1	1
Poorer	0.99 [0.95, 1.03]	0.97 [0.94, 1.01]
Middle	0.96 [0.91, 1.01]	0.92 [0.88, 0.95]*
Richer	0.93 [0.87, 1.01]	0.88 [0.83, 0.93]*
Richest	0.77 [0.68, 0.88]*	0.75 [0.68, 0.83]*
**Maternal age (in years)**		
<20	1	1
20–29	1.26 [1.18, 1.35]*	1.19 [1.12, 1.26]**
≥30	1.32 [1.21, 1.44]*	1.18 [1.09, 1.28]**
**Maternal educational status**		
No	1	1
Primary	1.10 [1.00, 1.21]*	1.09 [1.02, 1.18]*
Secondary	0.95 [0.84, 1.09]	0.99 [0.91, 1.09]
Higher	0.59 [0.50, 0.69]**	0.66 [0.60, 0.77]**
**Maternal employment status**		
Working	1.16 [1.10, 1.24]**	1.14 [1.08, 1.20]**
Not working	1	1
**Media exposure**		
No	1	1
Yes	1.03 [0.97, 1.10]	1.09 [1.05, 1.14]*
**Sex of household head**		
Male	1	1
Female	0.98 [0.94, 1.02]	0.98 [0.94, 1.02]
**Marital status**		
Married	1	1
Cohabiting	1.15 [1.05, 1.76]*	1.17 [1.07, 1.28]*
**Parity**		
Nulliparous	1	1
1–4	1.42 [1.29, 1.56]**	1.32 [1.22, 1.43]*
≥5	1.61 [1.43, 1.82]**	1.45 [1.33, 1.58]*
**Sexual autonomy**		
No	1	1
Yes	1.28 [1.15, 1.41]*	1.28 [1.17, 1.40]*

CI: Confidence Interval,

*<p-value<0.05,

** = p-value<0.01

The PAF findings showed that sexual autonomy had significant effect on IPV. About 19.82% of intimate partner violence was attributable to sexual autonomy (PAF% = 19.82%, 95% CI: 12.96, 26.15) (Table 5).

**Table 5:** Estimated population attributable risk and population attributable fraction of IPV among women in sub-Saharan Africa.

Variables	Adjusted PR	PAF%
Sexually autonomous		
No	1	
Yes	1.28 [1.17, 1.40]*	19.82 (12.96, 26.15

*PAF%: Population Attributable Fraction percent, PR: Prevalence Ratio

## Discussion

We examined the association between sexual autonomy and intimate partner violence among married and cohabiting women in sub-Saharan Africa. This study was worthwhile considering the global community’s focus on achieving gender equality by 2030, as echoed by the fifth Sustainable Development Goal (SDG) [20]. Overall, we found the prevalence level of sexual autonomy to be about three-fold that of intimate partner violence (88.79% vs 32.96%). The prevalence level of sexual autonomy found in our study is mixed, when compared with the prevalence reported in previous studies. For instance, while our prevalence level is consistent with Budu and colleagues’ 83.35% prevalence [39], it is higher than Aboagye and colleagues’ 73.0% prevalence [40]. This difference could be attributable to the countries and/or years of the dataset included in these studies. Interestingly, the relatively high prevalence of sexual autonomy we found, which has also been reported in previous studies [39, 40], contradicts the general belief of sexual subservience among women in settings where conservative sexual cultures are prominent [41–43].

Regarding intimate partner violence, our prevalence level of 32.96% is nearly consistent with the levels reported in previous studies among women in SSA [39, 40, 44]. The slight differences in the IPV levels could be attributable to differences in datasets, time periods and countries included in each of these studies. However, overall, the IPV prevalence levels reported in our study and previous studies in SSA are consistent with the global prevalence level of IPV among women [23]. In line with the results of previous studies among women in SSA, we found that IPV has a statistically significant association with sexual autonomy [40, 44], wealth status [40, 44] and maternal education [26, 40]. In the same vein, in support of previous studies, we found statistically significant association between IPV and media exposure [40, 44], maternal employment status [40] and age [40, 44].

In an era of globalisation when the boundaries of traditional sociocultural stereotypes are increasingly being challenged and pushed [45, 46], there is a need for context-specific family life education to change gender expectations and behaviours among men and women in SSA. Such education could help address misconceptions around feminism and autonomy among men and women––which may partly explain the association between sexual autonomy and IPV.

Despite the level of change in the sociocultural milieu of SSA, the subregion remains largely a patriarchal society [47, 48]. Against this backdrop, a progressive and well-managed change in gender expectations and behaviours would be preferable over a radical change. To this end, it is essential to plan, implement and periodically evaluate family life and community health education to conscientize women to practise sexual autonomy with discretion and men to accept and respect women’s autonomy [49]. This conscious and well-managed change is necessary as a breakdown in family relationships and IPV in SSA may be partly linked to what we would refer to as *misconceived feminism* among men and women alike. Feminism is not and should not be considered as a declaration of war against men. Rather, it is a noble course to be championed by both men and women, in reflection of *African feminism*–which recognises the role of men in women’s equality [50].

Furthermore, in line with previous studies [40, 44], we found a statistically significant association between wealth status and IPV. Our result showed that women from poorest households are likely to report twice the level of IPV prevalence reported among women from richest households. This result enunciates the ripple effect of poverty on social health and overall wellbeing. Socio-economic frustrations among men and women could become effective triggers of IPV with concomitant negative outcomes on women [50], children [51] and even men themselves [52]. Thus, using the Sustainable Development Goals as launching pads, government and non-governmental institutions and stakeholders could weaponize poverty eradication as an effective strategy against IPV.

One effective strategy for poverty eradication is funding and providing universal functional education for every member of society. In this regard, aside from conventional education, functional adult education could also be used as a strategy against both poverty and IPV [53]. Our result showed a statistically significant association between both maternal and husband education and IPV. Functional education––incorporating family life education––is an empowerment that could be protective against both poverty and IPV among men and women. On the one hand, this education could be an effective strategy for men (boys) and women (girls) to better manage gender relationships and prevent IPV [54]. On the other hand, it could also be a form of economic empowerment for men and women, which based on our findings, could half the likelihood of IPV occurrence.

Moreover, one key finding of our study, consistent with similar studies in SSA [40, 44], is the association between media exposure and IPV. We found that women who reported higher media exposure were also more likely to report a higher prevalence of experiencing IPV. This resonates with our earlier assertion about *misconceived feminism* which may be propagated in media content. Therefore, media contents relating to gender relationships and expectations must be circumspectly produced and consumed [55]. The media space, while an effective tool for education, entertainment, and enlightenment, should also not provide a platform for malicious content or content that could implicitly or explicitly promote IPV. Media content makers must not abuse the use of social media as a tool for free speech. Freedom also comes with a price—in this regard, the price to ensure that media contents encourage and instil mutual gender respect and harmonious living.

Another key finding of our study is a higher prevalence of IPV among cohabiting than married women. In this era of globalisation, it is important to protect and promote traditional cultural practices and values that are safe and health promoting. One of such safe traditional culture that is gradually losing traction in SSA is the practice of living together in a sexual relationship only with a man or woman to whom one is formally married to. Conversely, cohabitation is gradually gaining popularity in SSA [56]. With evidence suggesting higher IPV in such relationships, it may be prudent and proactive to encourage people entering into heterosexual relationships to formalise such relationships in marriages. Such formalisations could likely increase emotional and other forms of commitment, which could reduce, if not eradicate the likelihood of IPV.

### Strengths and limitations

It is worthy of note that robust analytical techniques were applied to high-quality national surveys of 27 countries in sub-Saharan Africa. All these surveys are representative of the included countries, hence the possibility of generalising our findings. Despite these outstanding strengths, the study has some limitations. First, there is the possibility of social desirability bias, leading to either under or over-reporting among the surveyed women. Besides, this is a cross-sectional study and as a result, causal inference is not permissible.

## Conclusion

This study has demonstrated that sexual autonomy is significantly associated with intimate partner violence, however, it does not necessarily act as a protective factor. This calls for further research to gain a deeper understanding of why sexually autonomous women stand a higher likelihood of intimate partner violence. Meanwhile, the study offers credence to some striking policy implications. Thus, there may be a need for more education on intimate partner violence targeting women’s partners. This can help secure the commitment of the perpetrators to become proponents of anti-intimate partner violence and further offer women the necessary support for them to attain their full fundamental women’s rights in all spheres of life. There is a need for the development of targeted policies and interventions in rural areas to address the higher tendency of IPV. These interventions could include awareness campaigns, support services, and community-based initiatives that challenge harmful gender norms and promote healthy relationships. As the study has indicated that women from wealthier households tend to have lower rates of IPV, addressing wealth disparities could aid in efforts to mitigate the prevalence of IPV. These recommendations, if prioritised, will go a long way to bolster the prospects of the included countries in their quest to achieve SDG 5 (gender equality and women empowerment), on gender equality by 2030.

## Supporting information

S1 Checklist STROBE statement—Checklist of items that should be included in reports of observational studies. (DOCX)

## References

[1] Bagarozzi DA. Enhancing intimacy in marriage: A clinician’s guide. Routledge; 2014.

[2] Czyżowska D, Gurba E, Czyżowska N, Kalus A, Sitnik-Warchulska K, Izydorczyk B. Selected predictors of the sense of intimacy in relationships of young adults. International journal of environmental research and public health. 2019 Nov;16(22):4447. doi: 10.3390/ijerph16224447 31766110 PMC6888334

[3] Friedman EJ. Gendering the agenda: The impact of the transnational women’s rights movement at the UN conferences of the 1990s. InWomen’s studies international forum 2003 Jul 1 (Vol. 26, No. 4, pp. 313–331). Pergamon.

[4] Memiah P, Opanga Y, Bond T, Cook C, Mwangi M, Fried J, et al. Is sexual autonomy a protective factor for neonatal, child, and infant mortality? A multi-country analysis. PLoS ONE. 2019;14(2): e0212413. doi: 10.1371/journal.pone.0212413 30794592 PMC6386489

[5] Jewkes R, Morrell R, Christofides N. Empowering teenagers to prevent pregnancy: lessons from South Africa. Culture, health & sexuality. 2009 Oct 1;11(7):675–88. doi: 10.1080/13691050902846452 19459086

[6] Rominski SD, Gupta M, Aborigo R, Adongo P, Engman C, Hodgson A, et al. Female autonomy and reported abortion-seeking in Ghana, West Africa. Int J Gynaecol Obstet. 2014;126(3):217–22. doi: 10.1016/j.ijgo.2014.03.031 24920181

[7] Potter JE, Stevenson AJ, Coleman-Minahan K, Hopkins K, White K, Baum SE, et al. Challenging unintended pregnancy as an indicator of reproductive autonomy. Contraception. 2019;100(1):1–4. doi: 10.1016/j.contraception.2019.02.005 30851238 PMC6919552

[8] Kantorová V. Unintended pregnancy and abortion: what does it tell us about reproductive health and autonomy?. The Lancet Global Health. 2020 Sep 1;8(9):e1106–7. doi: 10.1016/S2214-109X(20)30342-9 32710832 PMC7375788

[9] Willie TC, Callands TA, Kershaw TS. Intimate partner violence, sexual autonomy and postpartum STD prevention among young couples: a mediation analysis. Perspect Sex Reprod Health. 2018;50(1):25–32. doi: 10.1363/psrh.12050 29431903 PMC5996382

[10] Viswan SP, Ravindran TKS, Kandala N, Petzold MG, Fonn S. Sexual autonomy and contraceptive use among women in Nigeria: fndings from the Demographic and Health Survey data. Int J Women’s Health. 2017;9:581–90. doi: 10.2147/IJWH.S133760 28883744 PMC5574684

[11] Sougou NM, Bassoum O, Faye A, Leye MMM. Women’s autonomy in health decision-making and its efect on access to family planning services in Senegal in 2017: a propensity score analysis. BMC Public Health. 2020;20:872. doi: 10.1186/s12889-020-09003-x 32503492 PMC7275346

[12] Heidari S. Sexual rights and bodily integrity as human rights. Reprod Health Matters. 2015;23(46):1–6. doi: 10.1016/j.rhm.2015.12.001 26718991

[13] Dana-Sophia V. The human right to sexual autonomy. German Law Journal. 2021;22:703–17.

[14] Solanke B.L., Adetutu O.M., Sunmola K.A. et al. Multi-level predictors of sexual autonomy among married women in Nigeria. BMC Women’s Health 22, 114 (2022). doi: 10.1186/s12905-022-01699-w 35413895 PMC9003154

[15] Svanemyr J, Amin A, Robles OJ, Greene ME. Creating an enabling environment for adolescent sexual and reproductive health: a framework and promising approaches. Journal of adolescent health. 2015 Jan 1;56(1):S7–14. doi: 10.1016/j.jadohealth.2014.09.011 25528980

[16] Guilbert, N. Early marriage, women empowerment and child mortality: married too young to be a good mother; 2013. In Selected paper presented in the DIAL Development Conference" Institutions and Development" June 27th-28th.

[17] García-Moreno C, Pallitto C, Devries K, Stöckl H, Watts C, Abrahams N. Global and regional estimates of violence against women: prevalence and health effects of intimate partner violence and non-partner sexual violence. Geneva: World Health Organization; 2013.

[18] Krug EG, Mercy JA, Dahlberg LL, Zwi AB. The world report on violence and health. Lancet. 2002; 360: 1083–1088. doi: 10.1016/S0140-6736(02)11133-0 12384003

[19] Stöckl H, Devries K, Rotstein A, Abrahams N, Campbell J, Watts C, et al. The global prevalence of intimate partner homicide: a systematic review. The Lancet. 2013 Sep 7;382(9895):859–65. doi: 10.1016/S0140-6736(13)61030-2 23791474

[20] United Nations, Transforming our world: the 2030 agenda for Sustainable Development United Nations, 2015, A/RES/70/1.

[21] UN Comm. Elimin. Discrim. Against Women. 1992. CEDAW general recommendation no. 19: violence against women. Doc. A/47/38, United Nations, New York. https://www.refworld.org/docid/52d920c54.html

[22] Bagwell-Gray ME, Messing JT, Baldwin-White A. Intimate partner sexual violence: A review of terms, definitions, and prevalence. Trauma, Violence, & Abuse. 2015 Jul;16(3):316–35. doi: 10.1177/1524838014557290 25561088

[23] Sardinha L, Maheu-Giroux M, Stöckl H, Meyer SR, García-Moreno C. Global, regional, and national prevalence estimates of physical or sexual, or both, intimate partner violence against women in 2018. The Lancet. 2022 Feb 26;399(10327):803–13. doi: 10.1016/S0140-6736(21)02664-7 35182472 PMC8885817

[24] Niolon PH, Centers for Disease Control and Prevention. Preventing intimate partner violence across the lifespan: A technical package of programs, policies, and practices. Government Printing Office; 2017.

[25] Tessema ZT, Gebrie WM, Tesema GA, Alemneh TS, Teshale AB, Yeshaw Y, et al. Intimate partner violence and its associated factors among reproductive-age women in East Africa:-A generalized mixed effect robust poisson regression model. PLoS one. 2023 Aug 18;18(8):e0288917. doi: 10.1371/journal.pone.0288917 37594977 PMC10437948

[26] Kebede SA, Weldesenbet AB, Tusa BS. Magnitude and determinants of intimate partner violence against women in East Africa: multilevel analysis of recent demographic and health survey. BMC women’s health. 2022 Mar 17;22(1):74. doi: 10.1186/s12905-022-01656-7 35300675 PMC8928594

[27] Izugbara CO, Obiyan MO, Degfie TT, Bhatti A. Correlates of intimate partner violence among urban women in sub-Saharan Africa. PLoS One. 2020 Mar 25;15(3):e0230508. doi: 10.1371/journal.pone.0230508 32210457 PMC7094863

[28] Bachwenkizi J, Mohamed H, Funsan P, Rweyemamu D, Nelson W, Shao M, et al. Access to water sources and intimate partner violence against women in 26 Sub-Saharan African countries. Hygiene and Environmental Health Advances. 2023 Sep 1;7:100063.

[29] Ahinkorah BO, Dickson KS, Seidu AA. Women decision-making capacity and intimate partner violence among women in sub-Saharan Africa. Archives of Public Health. 2018 Dec;76(1):1–0. doi: 10.1186/s13690-018-0253-9 29423218 PMC5787915

[30] Mossie TB, Fenta HM, Tadesse M, Tadele A. Mapping the disparities in intimate partner violence prevalence and determinants across Sub-Saharan Africa. Frontiers in public health. 2023;11. doi: 10.3389/fpubh.2023.1188718 37448663 PMC10337829

[31] Rabin RF, Jennings JM, Campbell JC, Bair-Merritt MH. Intimate partner violence screening tools: a systematic review. American journal of preventive medicine. 2009 May 1;36(5):439–45. doi: 10.1016/j.amepre.2009.01.024 19362697 PMC2688958

[32] Ahinkorah BO. Intimate partner violence against adolescent girls and young women and its association with miscarriages, stillbirths and induced abortions in sub-Saharan Africa: evidence from demographic and health surveys. SSM-Population Health. 2021 Mar 1;13:100730. doi: 10.1016/j.ssmph.2021.100730 33511264 PMC7815812

[33] Ward CL, Harlow S. RESPecT and intimate partner violence: a cross-sectional study using DHS data in Kenya. BMJ open. 2021 Sep 1;11(9):e046069. 10.1136/bmjopen-2020-046069 PMC8424846 34493507

[34] Walters CN, Rakotomanana H, Komakech JJ, Stoecker BJ. Maternal experience of intimate partner violence is associated with suboptimal breastfeeding practices in Malawi, Tanzania, and Zambia: insights from a DHS analysis. International Breastfeeding Journal. 2021 Dec;16:1–2. 33602285 10.1186/s13006-021-00365-5 PMC7890985

[35] Rodriguez G, Elo I. Intra-class correlation in random-effects models for binary data. The Stata Journal. 2003 Mar;3(1):32–46.

[36] Merlo J, Chaix B, Ohlsson H, Beckman A, Johnell K, Hjerpe P, et al. A brief conceptual tutorial of multilevel analysis in social epidemiology: using measures of clustering in multilevel logistic regression to investigate contextual phenomena. Journal of Epidemiology & Community Health. 2006 Apr 1;60(4):290–7. doi: 10.1136/jech.2004.029454 16537344 PMC2566165

[37] Schiaffino A, Rodriguez M, Pasarin MI, Regidor E, Borrell C, Fernandez E. Odds ratio or prevalence ratio? Their use in cross-sectional studies. Gaceta Sanitaria. 2003: 1;17(1):70–4. 12605749 10.1016/s0213-9111(03)71694-x

[38] Barros AJ, Hirakata VN. Alternatives for logistic regression in cross-sectional studies: an empirical comparison of models that directly estimate the prevalence ratio. BMC medical research methodology. 2003 Dec;3(1):1–3. doi: 10.1186/1471-2288-3-21 14567763 PMC521200

[39] Budu E, Ahinkorah BO, Seidu AA, Hagan JE Jr, Agbemavi W, Frimpong JB, et al. Child Marriage and Sexual Autonomy among Women in Sub-Saharan Africa: Evidence from 31 Demographic and Health Surveys. Int J Environ Res Public Health. 2021;18(7):3754. doi: 10.3390/ijerph18073754 33916845 PMC8038468

[40] Aboagye RG, Dadzie LK, Arthur-Holmes F, Okyere J, Agbaglo E, Ahinkorah BO, et al. Intimate partner violence against married and cohabiting women in sub-Saharan Africa: does sexual autonomy matter? Reprod Health. 2022;19(1):79. doi: 10.1186/s12978-022-01382-1 35346246 PMC8962047

[41] Fladseth K, Gafos M, Newell ML, McGrath N. The impact of gender norms on condom use among HIV-positive adults in KwaZulu-Natal. South Africa PloS One. 2015;10(4):e0122671. doi: 10.1371/journal.pone.0122671 25853870 PMC4390283

[42] Lucea MB, Hindin MJ, Gultiano S, Kub J, Rose L. The context of condom use among young adults in the Philippines: implications for HIV prevention. Health Care Women Int. 2013;34(3–4):227–248. doi: 10.1080/07399332.2012.721414 23394323 PMC3578603

[43] Okafor UO, Crutzen R, Aduak Y, Adebajo S, Van den Borne HW. Behavioural interventions promoting condom use among female sex workers in sub- Saharan Africa: a systematic review. Afr J AIDS Res. 2017;16(3):257–268. doi: 10.2989/16085906.2017.1358753 28978291

[44] Ahinkorah BO. Polygyny and intimate partner violence in sub-Saharan Africa: Evidence from 16 cross-sectional demographic and health surveys. SSM Population Health. 2021; 13:100729. doi: 10.1016/j.ssmph.2021.100729 33511263 PMC7815814

[45] Folorunso CA. Globalization, Cultural Heritage Management and the Sustainable Development Goals in Sub-Saharan Africa: The Case of Nigeria. Heritage. 2021; 4(3):1703–1715. doi: 10.3390/heritage4030094

[46] Kaoma K. Contesting Global Culture: Globalization and Sexual Politics in Sub-Saharan Africa. In: Christianity, Globalization, and Protective Homophobia. Palgrave Macmillan, Cham. 2018.

[47] Baten J, de Haas M, Kempter E, Meier zu Selhausen F. Educational Gender Inequality in Sub-Saharan Africa: A Long-Term Perspective. Population and Development Review, 2021; 47: 813–849. doi: 10.1111/padr.12430

[48] Sikweyiya Y, Addo-Lartey AA, Alangea DO, Dako-Gyeke P, Chirwa ED, Coker-Appiah D, et al. Patriarchy and gender-inequitable attitudes as drivers of intimate partner violence against women in the central region of Ghana. BMC Public Health. 2020;20(1):682. doi: 10.1186/s12889-020-08825-z 32404153 PMC7222313

[49] Ghosh R, Chakravarti P, Mansi K. Women’s empowerment and education: Panchayats and women’s Self-help Groups in India. Policy Futures in Education. 2015 Apr;13(3):294–314.

[50] Stuhlhofer EW. Black, Female, and Divorced: A Discourse Analysis of Wangarĩ Maathai’s Leadership with Reflections from Naleli Morojele‘s Study of Rwandan and South African Female Political Leaders. Societies. 2022 Feb 9;12(1):23.

[51] Potter LC, Morris R, Hegarty K, García-Moreno C, Feder G. Categories and health impacts of intimate partner violence in the World Health Organization multi-country study on women’s health and domestic violence. Int J Epidemiol. 2021;50(2):652–662. doi: 10.1093/ije/dyaa220 33326019

[52] Wathen CN, Macmillan HL. Children’s exposure to intimate partner violence: Impacts and interventions. Paediatr Child Health. 2013;18(8):419–22. 24426794 PMC3887080

[53] Mbadugha EI. Intimate partner violence and sexual violence against women: any end in sight?. International Journal of Medicine and Biomedical Research. 2016 Apr 4;5(1):9–18.

[54] Campbell J.C. and Manganello J., 2006. Changing public attitudes as a prevention strategy to reduce intimate partner violence. Journal of Aggression, Maltreatment & Trauma, 13(3–4), pp.13–39.

[55] Slakoff DC, Aujla W, PenzeyMoog E. The role of service providers, technology, and mass media when home isn’t safe for intimate partner violence victims: best practices and recommendations in the era of CoViD-19 and beyond. Archives of sexual behavior. 2020 Aug 25:1–0. doi: 10.1007/s10508-020-01820-w 32844303 PMC7447204

[56] Odimegwu C, Ndagurwa P, Singini MG, Baruwa OJ. Cohabitation in Sub-Saharan Africa. Southern African Journal of Demography 2018; 18(1): 111–170.

